# Maintenance and Inspection of Fiber-Reinforced Polymer (FRP) Bridges: A Review of Methods

**DOI:** 10.3390/ma14247826

**Published:** 2021-12-17

**Authors:** Long Tang

**Affiliations:** School of Human Settlements and Civil Engineering, Xi’an Jiaotong University, 28 Xianning West Road, Xi’an 710049, China; tanglong@xjtu.edu.cn

**Keywords:** bridge monitoring, debonding, fiber-reinforced polymers, FRP, maintenance, non-destructive inspection

## Abstract

Fiber-reinforced polymers (FRPs) are materials that comprise high-strength continuous fibers and resin polymer, and the resins comprise a matrix in which the fibers are embedded. As the technique of FRP production has advanced, FRPs have attained many incomparable advantages over traditional building materials such as concrete and steel, and thus they play a significant role in the strengthening and retrofitting of concrete structures. Bridges that are built out of FRPs have been widely used in overpasses of highways, railways and streets. However, damages in FRP bridges are inevitable due to long-term static and dynamic loads. The health of these bridges is important. Here, we review the maintenance and inspection methods for FRP structures of bridges and analyze the advantages, shortcomings and costs of these methods. The results show that two categories of methods should be used sequentially. First, simple methods such as visual inspection, knock and dragging-chain methods are used to determine the potential damage, and then radiation, modal analysis and load experiments are used to determine the damage mode and degree. The application of FRP is far beyond the refurbishment, consolidation and construction of bridges, and these methods should be effective to maintain and inspect the other FRP structures.

## 1. Introduction

Fiber-reinforced polymers (abbreviation FRP) are compound materials [1,2,3,4]. Generally, FRP is composed of resin and fibers with high strength. The fibers are continuous and embedded in the resin. Thus, the resin is a matrix and plays the role of binder to hold and protects the fibers. The fibers act as reinforcing components, and the loads are transferred between the fibers through the resin. The intimal range of FRP application is evident in the aerospace, electric-car, high-speed-train and robot industries, for example. The technology of FRP manufacturing has advanced in recent years [4,5,6,7,8]. In particular, the pultrusion process has been developed to manufacture continuous and long FRPs. Such FRPs can effectively resist ultraviolet rays, chemical corrosion, the freeze-thaw cycle, the dry-wet cycle, high temperatures and humidity, and are light and high-strength [4,5,6,7,8]. In this way, FRPs are widely used in the field of architecture. According to the type of fiber, the FRPs applied in civil engineering are often aramid-FRP, basalt-FRP, carbon-FRP and glass-FRP. The resin often consists of vinyl ester, epoxy and polyester [4,5,6,7,8].

FRPs are now intensively used in the refurbishment and construction of bridges [4,5,6,7,8]. The decrease of the dead-weight of bridges and the increase of the load of bridges are important due to the demand for the increase of bridge span. Notably, when the span of a bridge is very large and the bridge is made of reinforced concrete, about eighty-five percent of the bridge load is dead-weight. The decrease of bridge’s dead-weight depends on light, high-strength and durabile materials. FRPs are an indispensable part of modern bridge structures [4,5,6,7,8]. FRP bridges are relatively light and constitute 30% to 60% of the weight of traditional structures. The advantages of FRP bridges are their strong tolerance to environmental corrosion, ease of construction and low cost of repair. Therefore, FRP bridges can be widely used as overpasses of highways, railways and streets. At the same time, FRP bridges also have important applications in rapid responses to emergencies, with significant economic and social benefits [4,5,6,7,8].

Some studies have reviewed the types of FRP applications in reinforced concrete structures [8,9,10,11,12]. Flexural application means that the boards, strips and fabrics of FRPs are pasted on a simply supported beam. Shear application is a technique including near-surface-mounted FRP plates and side bonding of FRP sheets. The technologies of torsion application are similar to those of flexural and shear application. The technology of axial application involves FRP composites that are spirally wrapped around concrete columns. The combined loading application is the system of near-surface-mounted FRP composite.

Bridges are often affected by factors such as climate, oxidation, corrosion and static and dynamic loads. Thus, damage to bridges is inevitable. The initial damage is the debonding of the surface layer of FRP reinforcement because the surface layer is the first to take the stress transmitted by the anchorage [6,7,10,11]. Then, the damage accumulates at the microstructure level of FRP reinforcement over time [6,7,10,11]. When the level of damages is higher than a certain threshold, the FRP reinforcement fails [6,7,10,11]. The damages of FRP reinforcement are joint failure, such as resin crack, resin transverse crack, interface debonding, delamination, fiber fracture and so on [6,7,10,11].

The components of bridges gradually age over time, threatening traffic safety. Therefore, it is necessary to maintain and manage bridges and take various technical measures to prolong their service life. The evaluation of material properties and the inspection of FRP structures have received a great deal of attention [12,13,14,15,16,17,18]. Here, the techniques for the maintenance and inspection of FRP bridges are reviewed. The aim is to summarize the details of techniques, in particular, to compare the cost, equipment and accuracy of these techniques of inspection. Thus, the proper techniques can be chosen easily.

## 2. Maintenance of FRP Structures

Bridge maintenance refers to regular maintenance, repair or reconstruction work. These measures can maintain the normal functions of bridges and their appendages, repair bridge damage and improve the service of bridges. According to the current, “Standard for maintenance of highway bridge and culvert” [19], bridge maintenance includes minor repairs, medium repairs, major repairs, reconstruction and special projects. The minor repair (daily maintenance) of a bridge refers to preventive maintenance of bridges and their affiliated structures and slight damage repairs. Medium repair refers to small engineering projects, including the regular repair and reinforcement of the wear and damage in bridges and affiliated structures. Major repair refers to the periodical and comprehensive repair of considerable damage in bridges and affiliated structures to recover the health level of the design standard, or the improvement of parts and addition of more structures within the scope of the technical grade to improve the capacity of bridge. Reconstruction refers to the larger engineering projects that improve the technical grade, or reconstruction of some parts to improve the capacity, load and flood discharge.

The service of a bridge exceeds the required level during operation, and the cost of maintenance and the impact on the environment are minimized, which is the goal of bridge maintenance [20,21,22,23,24,25,26,27,28,29]. Specifically, the bridge is clean; the deck is solid and flat; the cross slope is moderate; the connection of the bridge head is smooth; the drainage is smooth; the structure is complete; and auxiliary facilities such as signs, markings and lamps are intact [20,21,22,23,24,25,26,27,28,29]. The maintenance of the FRP structure mainly includes five categories (Table 1).

### 2.1. Sunscreen and Waterproof

The surface of the FRP will lose luster and color after long-term exposure. In addition, after long-term rain erosion, water can penetrate into the material along the microscopic chink at the interface between the fiber and resin, which weakens the bonding force between fiber and resin, leads to material aging, and thus damages the mechanical properties of the material. Therefore, it is necessary to paint anti-corrosion materials on the surface of FRP components and check them frequently when FRP bridges are in hot and humid environments [20,21,22,23,24,25].

### 2.2. Repairing Surface Damage

The resin layer on the surface of the FRP component is thin, and resin peeling, deep scratching, fiber exposure and other damage will occur after collision, impact and friction. Therefore, it is necessary to frequently inspect the surface of FRP components and repair the damage in time. Otherwise, rainwater will infiltrate, expand pores and accelerate material damage. In addition, the ancillary facilities (e.g., parapet, sidewalk and lamp) that are made of FRP materials are often bumped, so it is necessary to set anti-collision devices to protect these FRP facilities.

The local damage of the FRP bridge can be repaired with fiber cloth and resin [20,21,26,27]. The procedure is as follows: (a) remove the loose resin and fiber from the damaged part, and then clean and dry the surface; (b) cut the fiber cloth according to the size of the surface; (c) blend the resin; and (d) apply the resin to paste fiber cloth.

### 2.3. Fire Prevention

FRP materials are thermosetting and may be vitrified under high temperatures and fire. Such vitrification will degrade the properties of materials and seriously endanger the stabilization of bridge structures. Therefore, it is necessary to clean up the combustible materials on and around the bridge in time [6,7,10,11].

### 2.4. Repairing the Debonding Part and Crack

Due to stress concentration and adhesive layer aging, delamination and adhesive layer debonding often occur between the FRP layers, FRP members and the interface between the FRP members and concrete. These changes can reduce the integrity of structures.

FRPs are brittle, so cracks usually appear in the area where stress is concentrated, such as bolt holes and corner joints of the profile. When the material cracks, the damage will deteriorate from the crack position.

Therefore, it is necessary to periodically check the delamination and debonding of FRP bridges, check whether there are cracks in the area of stress concentration and make repairs on time [10,11,28,29]. There are many check-related methods, and an important part of this review is nondestructive inspections of FRP bridges.

## 3. Nondestructive Inspections of FRP Bridges

Compared with metal, the advantages of FRP materials are their high strength, stiffness, radiation resistance and easy operation. However, due to the mechanical characteristics of FRP bridges, there are pores, impurities and delamination [6,7]. Thus, a monitoring system is crucial to locating and identifying damage as well as diagnosing structures. Furthermore, the sensors that receive and convert signals in the monitoring system must be appropriate, and the data analyses for judging the abnormal signal must be correct.

Previous studies have shown that the integrity of an FRP structure is strongly affected by the connection between the fiber and adhesive layer and the connection between the composite and impregnated resin. At present, a variety of nondestructive inspection techniques have been used to evaluate and identify the performance of materials, components and structures. In addition, FRP materials have been used in aircraft and ship structures for a long time, so the nondestructive inspections used in the fields of aviation and navigation can be used for the inspections of FRP bridges. Some methods, such as visual inspection, dragging-chain and knock methods, do not need to use special equipment. Other methods, such as thermal imaging, acoustic emission, ultrasonic waves, radiation, modal analysis and load tests, are more complex and require specific equipment [6,7,12,13,14,15,16,17,18]. According to the analysis of previous research results [30,31,32,33,34,35,36,37,38,39,40,41,42,43,44,45,46,47,48,49,50,51,52,53], the scope of the applications and functions are different among these methods (Table 2).

### 3.1. Visual Inspection

Visual inspection is the simplest and most widely used method. Visual inspection combined with some simple tools, such as flashlights, rulers and mirrors, can quickly detect damage in bridge structures. The disadvantage of visual inspection is that it can only detect the damage on the surface; however, it can neither quantify the damage nor detect the internal defects of the structures. Therefore, when cracks, delamination, discoloration, holes and deformation are found by visual inspection, further inspections through precise methods are necessary [30].

### 3.2. Knock

Inspectors use coins, hammers or electronic hammering units to strike the surface of FRP members, and they then detect delamination and holes by identifying the sounds. Inspectors also drag the iron chain on the bridge deck and locate the potential delamination through the change of sound. Knock combined with visual inspection can effectively identify most damage in FRP bridges. Alampelli [31] knocked the bottom of the bridge with a rubber hammer and found holes in the bottom of the FRP slab bridge deck. Rosenboom [32] found that visual inspection and knock can effectively identify the bond quality between FRP materials and concrete.

### 3.3. Thermal Imaging

The mechanism of thermal imaging is that the defects under the surface of the FRP component will affect the thermal flow in the material. The heat source is set on one side of the bridge deck, and the temperature is measured by the thermal imaging camera on the other side. According to the change in temperature, defects such as delamination, debonding, impact damage, moisture absorption and holes are determined. Thermal imaging can be natural or artificial.

The heat source of passive thermal imaging is the natural temperature. This method only allows for qualitative detection and can identify potential defects by detecting abnormal temperatures. The heat source of active thermal imaging is external and uniform. Debonding, cracking, impact damage, water accumulation and other defects can affect the thermal properties of materials. The area without defects can conduct heat more effectively than the area with defects. The absorption or reflection of heat can create a thermal gradient, and this gradient can be observed by a thermal imager. Because a thermal gradient can be caused by nonuniform heating, the heat source of active thermal imaging should be implemented on a surface that can be heated evenly.

Hag-Elsafi et al. [33] and Taillade et al. [34] successfully applied thermal imaging to detect the bond between FRP and concrete. Halabe et al. [35] used a digital thermal imager to detect defects under the surface of an FRP bridge deck and debonding between the wearing layer and bridge deck. The internal temperature of the bridge can be measured by a thermal sensor. Teng et al. [36] installed thermal sensors on the glass fiber-reinforced polymer (GFRP) concrete columns of expressway bridges to monitor the temperature difference between FRP and concrete and found possible debonding damage.

At present, thermal imaging is often used for the qualitative detection of damage in FRP structures. The results of laboratory tests and field tests show that thermal imaging is effective in the nondestructive testing of FRP strengthened concrete members and FRP bridge decks. This method can also be used to detect the quality of FRP members in the pultrusion process, installation process and service process. Notably, thermal imaging is probably ineffective in detecting thick FRP members.

### 3.4. Acoustic Emission

Acoustic emission is nondestructive and is used to detect the integrity of FRP structures. The mechanism of this method is the change in the intensity of the sound signal. Due to the rapid release of energy, a stress wave is generated when the material is loaded. The stress wave radiates from the wave source and is then recorded by the sensor arranged on the surface of the material. Usually, piezoelectric sensors are used to detect acoustic emission. When the stress wave is transmitted to the sensor, the crystal will produce an output signal due to the pressure. When the intensity of the signal is higher than the threshold that is set, the instantaneous signal is recorded as an impact. In the FRP structure, acoustic emission can be caused by matrix cracking, fiber debonding, delamination, fiber pullout and fiber fracture. The waves generated by fiber fracture can release high energy, and thus, the intensity of the acoustic emission signal is high. In contrast, the acoustic emission signal produced by matrix cracking and fiber matrix debonding is weak. The duration of the acoustic emission signal is longer, and the intensity is moderate.

The equipment for this inspection consists of a piezoelectric sensor, a coupling agent, multichannel data acquisition equipment and highly integrated analysis and data acquisition software. Gostautas et al. [37] successfully completed the evaluation and detection of six GFRP bridge decks that have various full-scale cross sections.

Some acoustic emission signals are false, so it is important to judge the authenticity of the signal. There are many reasons for false signals, such as mechanical friction, leakage, liquid flow, vibration, wind-induced vibration, rain, snow, hail and thermal expansion under sunlight. Additionally, it is also very common for bearing sliding to produce false signals.

### 3.5. Ultrasonic Waves

Ultrasonic wave inspection introduces high-frequency stress waves into the structure to detect defects or changes in material properties. Pulse reflection is the most commonly used method, and the energy of sound is introduced into the material as waves. When the waves encounter discontinuities (such as cracks) in the transmission path, the partial energy will be reflected back from the defects. The reflected wave is recorded by the sensor, converted into an electronic signal and then displayed on the screen. The location and size of the defect can be determined. This method is easy to operate, and common ultrasonic instruments can be used.

The equipment for ultrasonic waves is a converter, a pulse generator, a receiver/amplifier and a screen. This method has been used to detect debonding failure of concrete members strengthened by FRP [39] and the failure of iron bridges by strengthened FRP [40]. Muhmouda et al. [41] used surface acoustic waves (SAWs) to detect the interface degradation of concrete members strengthened by carbon fiber-reinforced polymer (CFRP) and suggested that this method be used to monitor the structures of concrete bridges strengthened by FRP. The operator should master professional cognition to carry out the inspection and interpret the test data. In addition, the cost of this method is high.

### 3.6. Radiation

Operators use X-rays or gamma rays to penetrate a component to detect defects. The radiation source is arranged on one side of the component, and the screen is arranged on the other side. The ability to absorb rays varies between defects in FRP bridge decks, such as delamination, and healthy parts. The location of the defect can be displayed on a radiographic film or a computer screen.

This method cannot capture the three-dimensional characteristics of defects. Alternatively, when the relative positions of defects, radiation source and film are appropriate, the method can provide high-resolution images of defect planes and can detect delamination and debonding. Therefore, it is necessary to adjust the relative positions of defects, radiation sources and films.

This method has been applied to the defect detection of composite laminates and sandwich plates [42,43]. The disadvantages of this method are the threat of radiation to the health of operators and the high cost. This method should be further improved before it is applied in civil engineering.

### 3.7. Ground Radar Wave

Ground penetrating radar projects electromagnetic waves into materials. When an electromagnetic wave encounters discontinuous defects, the waves produce reflected pulses. Discontinuous defects can be the boundary of materials, the interface of different media and delamination or debonding below the surface of materials. The position of the discontinuity can be determined by the amplitude of the reflection wave and the corresponding reflection time.

This method is effective in detecting damage under the surface of laminated materials (such as FRP composites), such as debonding between the wearing layer and FRP bridge deck and delamination of the flange of the FRP bridge deck. Compared with thermal imaging, ultrasonic waves and ground-penetrating radar waves have a stronger penetration ability, so they can detect deep defects and the degradation of concrete.

### 3.8. Microwaves

The frequency of electromagnetic energy ranges from several hundred MHz to several hundred GHz. Such high-frequency waves can penetrate thick FRP materials to determine the transmission and distribution of electromagnetic waves.

This method has been used to detect debonding, delamination and holes, in FRP concrete members. Important scale information, such as the spatial resolution, location and size of the debonding area, can be determined by the microwave method. Li and Liu [44] and Buyukozturk et al. [45] used microwaves to detect holes between FRP and concrete. Aboukhousa and Qaddoumi used electromagnetic waves [46] to inspect a composite and found that reflected waves can indicate debris under the surface of a composite plate with five layers and 45.6 mm thickness.

### 3.9. Optical Fiber Sensing

Optical fiber sensing is widely used to detect the damage of intelligent structures in compound materials. This method is nondestructive, and the equipment is small and highly sensitive. An optical fiber is composed of hollow quartz glass, a photoconductive coating and a plastic outer protective layer. According to the law of refraction, light will only be reflected in the hollow glass. There are various optical fiber sensors, such as intensity sensors, spectrum sensors and interference sensors.

Researchers embed optical fiber sensors in FRP bars so that FRP materials are intelligent and can complete the detection or act as sensors by themselves. Kaamkarov et al. [47] embedded Fabry Perot and Bragg grating fiber optic sensors in pultruded GFRP and CFRP tendons. Thus, the whole FRP bar is the sensor. This method can be used to detect the health of prestressing cables. Sim et al. [48] studied the tensile strength and pullout performance of Bragg grating fiber optic sensors in GFRP bars and found that hybrid GFRP bars can be reinforcement materials for concrete structures and can perform intelligent monitoring.

An optical fiber is pasted or buried on the interface between layers of composite. Thus, the optical fiber is placed in the composite laminate, and the epoxy glue can protect the optical fiber. The strain of the material in the bonding surface of FRP concrete can be measured by this method, and the unloading recorded by the sensor can indicate the debonding of the surface between FRP and concrete bonding. Bonfigliol and Pascal [49] used optical fiber sensors to measure the strain on the concrete surface in the debonding area of the interface between FRP and concrete. Zhu et al. [50] arranged a sensor on the surface between an FRP pipe and concrete to measure the strain and cracks in the concrete.

Optical fiber sensors can be embedded into materials or interfaces between different materials during material production and bridge construction, and it is easy to combine optical fiber sensors with composite materials. Laylor and Kachlakev [51] used Bragg grating sensors to detect the durability of concrete beams strengthened by FRP in a Horsetail Falls bridge, Oregon, USA. Watkins et al. [52] used an optical fiber network composed of interference sensors to monitor the static and dynamic strains of a concrete bridge strengthened with FRP in Missouri, USA. The expectations of the design, finite element calculation results and measurement results are consistent. Fiber optic sensors are also widely used to monitor the construction of new FRP bridges and the reinforcement of FRP bridges in Canada.

### 3.10. Modal Analysis and Load Experiment

The mechanism of this method is the difference in the response to vibration or load. When an FRP bridge ages, the overall stiffness changes, resulting in the response to vibration or load changes. This change is used to evaluate the health of the bridge. Several acceleration sensors are arranged on the bridge, and then a predetermined load is applied on the bridge to determine the modality and vibration of the structure. The difference in information between the measured modal and initial theoretical modalities can be used to evaluate the degradation or damage of FRP bridges. Alampelli [31] used modal analysis to detect the health of the first FRP bridge in New York state. Guan et al. [53] arranged acceleration sensors on the FRP viaduct to collect vibration information and carried out modal analysis to determine the long-term performance of bridges.

When the load experiment is carried out, a strain gauge, accelerometer and displacement meter are set on the bridge structure to apply the preset load, and then the strain and displacement are measured. The severity of strain and displacement are used to evaluate the performance of the bridge. Load experiments should be conducted every period (such as 1–2 years) to detect the damage of the bridge structure over time.

## 4. Conclusion and Further Studies

The damages in the bridges with FRP structures are inevitable. For visual inspection, knock and dragging-chain methods, the equipment is simple, and the required professional skill is moderate. Alternatively, for thermal imaging, acoustic emission, radiation, modal analysis and field load tests; special and expensive test equipment; and more complex skills are needed. Therefore, the two groups of methods can be combined. First, a simple method is used to determine the potential damage, and then radiation, modal analysis and load experiments are used to determine the damage mode and degree. FRP materials have been a universal part of modern structures and can contribute to prolonging the service life of old structures, such as seismic reconstruction, the reinforcement of concrete structures, the maintenance of metal and wood beams and the repair of historical sites. The methods involved in actions related to inspection of FRP bridges can be applied to inspecting those structures with FRP materials. I share the view of Naser et al. that the application of sensing devices will provide insight into the long-term behavior of concrete structure strengthened by FRP materials [8], which should be the main method for inspecting FRP in the future with the progress of sensing device manufacturing technology and the reduction of price.

The maintenance and inspection of FRP bridges is a large and important specialization, and it has received a great deal of attention. However, although many studies have focused on the techniques of maintenance and inspection, few of them have demonstrated the conditions for the applications of these techniques. In particular, do the characteristics of bridges (such as span, height), uses (such as cross-sea and cross-railway bridges for automobiles, cross- street bridge for pedestrian passage), and environments where bridges are sited (such as temperature, humidity) influence the accuracy of techniques? What frequency and interval are proper for the maintenance and inspection of FRP bridges? Moreover, is it necessary to implement inspection during the construction of FRP bridges? Comparing the accuracy of various detection methods and linking machine learning with the mechanism of the detection is crucial for developing the intelligent inspection of FRP structure damage.

## Figures and Tables

**Table 1:** The Maintenance of FRP Bridge.

Name	Aim	Method
Sunscreen and waterproof	Reducing the rate of aging	Painting anti-corrosion materials on the surface
Repairing surface damage	Protecting FRP facilities	Pasting fiber cloth on facilities by resin
Fire prevention	Eliminating accidents	Cleaning bridge
Repairing debonding parts and cracks	Reinforcing and protecting the area in which stress concentrates	Replacing damaged materials with new ones

**Table 2:** Characteristics of Nondestructive Inspections of FRP Bridges.

Name	Equipment	Cost	Accuracy
Visual inspection [30]	Flashlight, mirror and ruler	Low	Low
Knock [31,32]	Coins, hammers, electronic hammer and iron chain	Low	Low
Thermal imaging [33,34,35,36]	Natural or artificial heat source, thermal imaging camera	Low	Natural heat source, low; artificial one, medium
Acoustic emission [37,38]	Piezoelectric sensor, multichannel data receiver	Medium	Medium
Ultrasonic wave [39,40,41]	Pulse generator, wave amplifier, and screen	Medium	Medium
Radiation [42,43]	Radiation source, screen	Medium	Low (still in its experimental stages, immature)
Ground radar wave [38]	Ground penetrating radar, receiver	Medium	Medium
Microwave [44,45,46]	Electromagnetic wave transmitter, wave amplifier	High	High
Optical fiber sense [38,47,48,49,50,51,52]	Optical fiber sensor	High	High
Modal analysis and load experiment [31,53]	Strain gauge, accelerometer	High	High

## Data Availability

Data sharing not applicable.
